# A Chinese family with progressive childhood cataracts and IVS3+1G>A *CRYBA3/A1* mutations

**Published:** 2010-11-09

**Authors:** Yanan Zhu, Xingchao Shentu, Wei Wang, Jinyu Li, Chongfei Jin, Ke Yao

**Affiliations:** Eye Center of the 2nd Affiliated Hospital, Medical College of Zhejiang University, Hangzhou, China

## Abstract

**Purpose:**

To characterize the disease-causing mutations in a Chinese family with progressive childhood cataracts.

**Methods:**

Family history and clinical data were recorded. Direct gene sequencing together with multi-point linkage analysis using microsatellite markers flanking the gene was applied to identify the disease-causing mutation.

**Results:**

Lens examination in the affected members revealed childhood cataracts along with progressive developing fetal nuclear lactescent cataracts with ‘Y’ sutural opacities, and also progressive developing peripheral cortical opacities. Direct gene sequencing showed a G>A transition at the donor splice site of intron 3 (IVS3+1 G>A) of the βA1/A3-crystallin gene (*CRYBA3/A1*) in this Chinese autosomal dominant childhood cataract family, and the maximum heterogeneity logarithm of odds (HLOD) score obtained by multi-point analysis was detected at marker locus D17S1800 (HLOD=3.005; α=1.000).

**Conclusions:**

To our knowledge, this is the first report of a phenotype of progressive nuclear and cortical cataracts related to the *CRYBA3/A1* mutation IVS3+1 G>A. This finding expands the spectrum of cataract phenotypes caused by the IVS3+1 G>A mutation of *CRYBA3/A1*, confirms the phenotypic heterogeneity of this mutation and suggests the mechanism that influences the cataractogenesis in different ethnic backgrounds.

## Introduction

Childhood cataracts are a cause of childhood blindness worldwide. This disease accounts for more than 1 million blind children in Asia and about 10% of the childhood blindness worldwide [1]. Childhood cataracts are also a clinically and genetically heterogeneous disorder in which the phenotype varies considerably between and within families [2]. From an etiological point of view, genetic mutation might be the most common cause, especially for bilateral cataracts. All three forms of Mendelian inheritance have been observed, and the most frequently seen in non-consanguineous populations is autosomal dominant (AD) transmission. At present, over 26 out of the 39 mapped loci for isolated congenital or childhood cataracts have been associated with mutations in specific genes [3]. Of the cataract families in which the mutant gene is known, approximately half have crystallin mutations, including the αA-crystallin gene (*CRYAA*), αB-crystallin (*CRYAB*), *CRYBA3/A1*, βA4-crystallin (*CRYBA4*), βB1-crystallin (*CRYBB1*), βB2-crystallin (*CRYBB2*), γC-crystallin (*CRYGC*), γD-crystallin (*CRYGD*) and γS-crystallin (*CRYGS*) [3-10], approximately one quarter have connexin mutations in Connexin 46 (*GJA3*) and Connexin 50 (*GJA8*) [11,12], with the remainder being divided among the genes for heat shock transcription factor-4 (*HSF4*) [13], aquaporin-0 (*AQP0, MIP*) [14,15], beaded filament structural protein-2 (*BFSP2*) [16], paired-like homeodomain 3 (*PITX3*) [17], chromatin modifying protein 4B (*CHMP4B*) [18], and EPH receptor A2 (*EPHA2*) [19]. Most of the mutations detected in these genes are missense and nonsense mutations [3]. Among them, *CRYAA* [20], *CRYBA3/A1* [21], *CRYBB2* [22], *CRYGC* [23], *CRYGD* [24], *GJA3* [25,26], *GJA8* [26], *BFSP2* [27], *MIP* [28], and *HSF4* [29] have been reported to be related to nuclear cataracts.

The *CRYBA3/A1* mutation was first identified as a cause of cataracts in a pedigree with autosomal dominant zonular cataracts [8]. To date, three mutations have been reported in *CRYBA3/A1*, IVS3+1 G>C [30], IVS3+1 G>A [8,21,31], and 279delGAG [32,33]. IVS3+1 G>A has been observed only in the Indian, Australian, and Chinese populations. In the Chinese population, this mutation is related to posterior polar cataracts [34].

In this study, we identified a Chinese family with *CRYBA3/A1* IVS3+1 G>A by genetic analysis. To our knowledge, this is the first report to relate this mutation site with progressive childhood cataracts characterized by opacities in the fetal nucleus and peripheral cortex.

## Methods

### Family data and genomic DNA preparation

**Figure 1:**
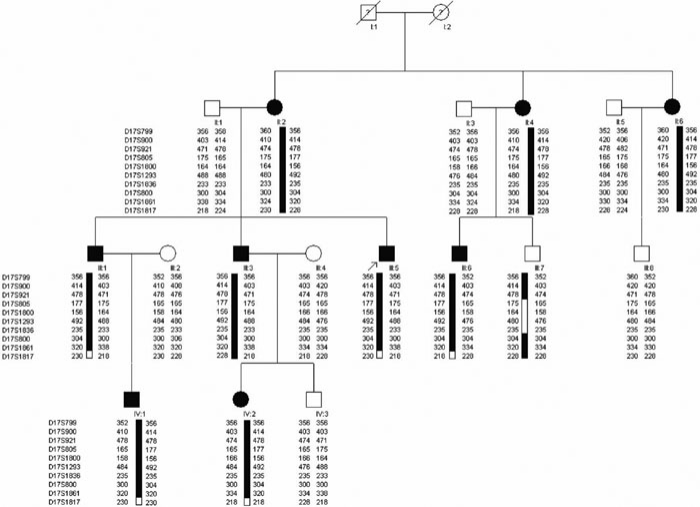
Pedigree of the proband. The black symbols indicate individuals with a diagnosis of congenital cataract performed by genetic analysis. The arrow indicates the proband. All of the members shown in this figure except I:1 and I:2 took part in this project. The haplotype markers are shown at the left of each generation. The black and white bars depict the disease and non-disease associated haplotype, respectively. Haplotype analysis identified the causative gene as being between D17S921 and D17S800 on 17p12–21.2.

A four-generation family with autosomal dominant childhood cataracts was ascertained through the Eye Center of the 2nd Affiliated Hospital, Medical College of Zhejiang University, Hangzhou, China, and this study was approved by the Zhejiang University Institutional Review Board. Appropriate informed consent was obtained from all participants and the study protocol followed the principles of the Declaration of Helsinki. Seventeen individuals (9 affected and 8 unaffected) from the family took part in the study (Figure 1). The affected status was determined by a history of cataract extraction or ophthalmologic examination, including visual acuity, slit lamp, and fundus examination. The phenotypes were documented by slit lamp photography.

### Genomic DNA preparation

We collected blood specimens (5 ml) in EDTA and extracted the genomic DNA from the peripheral blood leukocytes of the available family members using the Simgen Blood DNA mini kit (Simgen, Hangzhou, China).

### Mutation screening

We used the functional candidate gene analysis approach. Gene specific PCR primers for *CRYAA*, *CRYBA3/A1*, *CRYBB1*, *CRYBB2*, *CRYGC*, *CRYGD*, *GJA3*, *GJA8*, and *MIP* were designed flanking each exon and intron-exon junction. The cycling conditions for PCR were as follows: 95 °C preactivation for 5 min, 10 cycles of touchdown PCR with 1 °C down per cycle from 60 °C to 50 °C, followed by 25 cycles with denaturation at 95 °C for 25 s, annealing at 55 °C for 25 s and extension at 72 °C for 40 s, then finally extension at 72 °C for 10 min. Each reaction mix (25 µl) contained 50 ng of genomic DNA, 10× PCR buffer,1.5 mM MgCl_2_, 0.2 mM dNTPs, 5 µmol each of sense and antisense primers (Table 1) and 2.5U of Taq DNA polymerase (Sangon Biotech, Shanghai, China). Thermal cycling was performed under suitable conditions using a C1000^TM^ 48 well thermal cycler (Bio-Rad, Hercules, CA). PCR products were isolated by electrophoresis on 1.5% agarose gels and sequenced using the BigDye Terminator Cycle sequencing kit V 3.1(Applied Biosystems, Foster City, CA) on an Applied Biosystems PRISM 3730 Sequence Analyzer, according to the manufacturer’s directions.

**Table 1:** Primers used in polymerase chain reaction of *CRYBA3/A1*.

**Name**	**Prime Sequence(5′-3′)**	**Product length (bp)**
Exon1F	AGCAAGCTGAGCCACCAAAG	308
Exon1R	GCTGTCTTCCGCCAGAGTTC	
Exon2F	TCGTGTGTGCTCTGTCTTCC	205
Exon2R	CCCCTACAAACTGGGGTTTT	
Exon3F	CATCAGGCATCCCAGGCTACA	333
Exon3R	TCCTTCTTCCCCTATCCCCAC	
Exon4F	CGTCAACTCATTCCTCAACTCT	464
Exon4R	CAGGCTTAGAGAAGAAAGTGATGT	
Exon5F	TTTCTCACAAATCTGTTGCCTTA	340
Exon5R	CAAAGTAACTCCTGAGGTTGCA	
Exon6F	AGGCTCAGGTTTTGGGGTAT	471
Exon6R	ACTCCAGCCTGAGCAACAAG	

### Genotyping

We performed a partial genome scan in the vicinity of the *CRYBA3/A1* locus, and chose the 10 fluorescent short tandem repeat polymorphic markers for this locus which are shown in Figure 1. Multiplex PCR was performed in a 20 μl reaction mixture containing 10 ng of genomic DNA, 0.3 mM of each dNTP, 0.1 μM each of forward and reverse primers, 1 U HotStarTaq polymerase (Qiagen, Hilden, Germany), 3.0 mM MgCl_2_, and 1× HotStarTaq buffer. Samples were incubated in a thermocycler for 15 min at 95 °C and 20 s at 94 °C; the annealing temperature was programmed to initiate at 65 °C for 40 s and decrease 0.5 °C every cycle; then 68 °C for 2 min, for 11 cycles; followed by 94 °C for 20 s, 59 °C for 40 s, 68 °C for 2 min, for 24 cycles; a final extension at 60 °C for 1 h was performed. The PCR products were appropriately pooled and an aliquot was loaded onto a 5% standard denaturing polyacrylamide gel and run in an Applied Biosystems 3130xl Genetic Analyzer. The size of each allele was determined on the basis of an internal size standard (GeneScanTM −500 Liz Size Standard, Applied Biosystems) in each lane, and results were analyzed using the Applied Biosystems GeneMapper 4.0.

### Linkage analysis and haplotyping

Multi-point linkage analysis was calculated using Merlin. A gene frequency of 0.0001 and penetrance of 100% were assumed. Microsatellite markers, allele frequencies, and recombination distances between the marker loci were based on the Marshfield database and the UCSC database. Family and haplotype data were processed using Cyrillic software (version 2.1; Cyrillic, Oxfordshire, UK).

## Results

### Clinical evaluation

**Figure 2:**
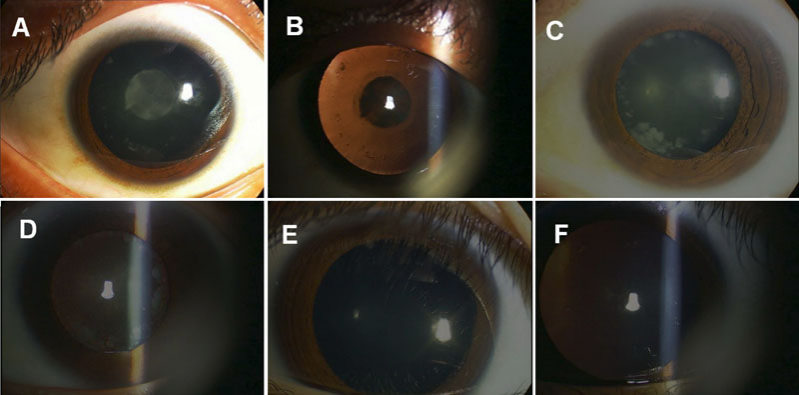
Photographs of the eyes of the family members with congenital cataracts. **A**: The left eye of proband III:5. Diffuse illumination shows a fetal nuclear lactescent cataract with ‘Y’ sutural opacities and mild peripheral cortical opacities. **B**: Retroillumination showing the left eye of proband III:5. **C**: The right eye of II:2. Diffuse illumination shows denser peripheral cortical opacities, but the opacity of nucleus is almost the same as III:5. **D**: Retroillumination showing the right eye of proband II:2. **E**: The left eye of IV:1. Diffuse illumination shows only mild nuclear and sutural opacities. F: Retroillumination showing the left eye of proband IV:1.

We identified a four-generation Chinese family with a clear diagnosis of autosomal dominant childhood cataracts. Most affected individuals noticed their visual impairments before the age of twenty, and then their visual acuity decreased gradually until surgery was required to restore their visual function before the age of 40 (Figure 1). Opacity of the lens was bilateral in all of the affected individuals. The proband, who was a 28 year-old, had nuclear lactescent cataracts, ‘Y’ sutural opacities, and dot-like peripheral cortical opacities. His clinical features were almost the same as his brother (III:3). The affected member II:2, who was the mother of the proband, had more severe, curd-like cortical opacities, but her nuclear opacities were not much different from those of her sons. In the childhood stage before the age of ten, like IV:1, who was a 9 year-old, there was no opacity in the peripheral cortex, but there were mild opacities in the nucleus which had been first identified when he was three years old (Figure 2). After the age of 10, like the case of IV:2, who was a 12 year-old, certain fine, sand like opacities developed. The clinical evaluation of the affected individuals is provided in Table 2. Before surgery, the affected members had a visual acuity which ranged from finger counting to 20/100. After surgery, all the patients achieved a distance visual acuity of 20/25 to 20/20. There was no family history of other ocular or systemic abnormalities.

**Table 2:** Clinical evaluation of affected individuals.

**Affected individual**	**Age at presentation**	**Age at cataract surgery**	**Phenotype**
II:2	55	55	Nuclear lactescent cataract, ‘Y’ sutural opacities, severe curd-like peripheral cortical opacities
II:4	48	36	Had cataract surgery 12 years ago
II:6	45	35	Had cataract surgery 10 years ago
III:1	35	30	Had cataract surgery 5 years ago
III:3	33	28	Nuclear lactescent cataract, ‘Y’ sutural opacities, fine sand-like peripheral cortical opacities
III:5	28	28	Nuclear lactescent cataract, ‘Y’ sutural opacities, fine sand-like peripheral cortical opacities
III:6	25	22	Had cataract surgery 3 years ago
IV:1	9	No surgery	Mild nuclear opacities with sutural opacities
IV:2	12	No surgery	Nuclear opacities with sutural opacities, a few dot-like cortical opacities

### Genetic analysis

**Figure 3:**
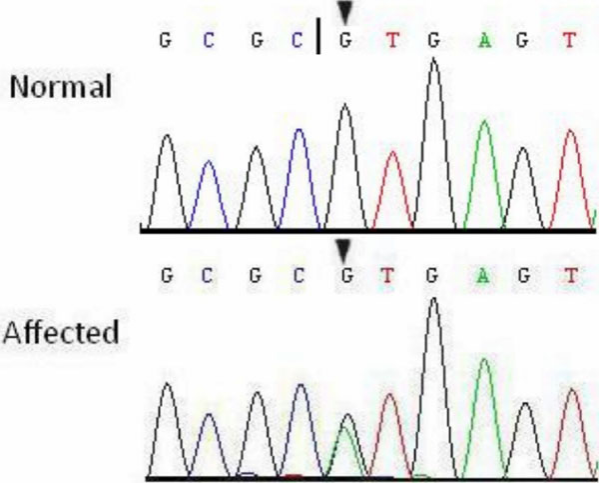
Partial DNA sequence of *CRYBA3/A1* from one normal and one affected individual, showing a heterozygous mutation (IVS3+1 G>A) in the third canonical GT site of *CRYBA3/A1* (black triangles). The black vertical line denotes the normal exon 3-intron 3 donor splice site.

Through gene sequencing we identified a single base substitution in the donor splice site of intron 3 in *CRYBA3/A1* (IVS3+1 G>A) which cosegregated with all affected individuals, whereas this heterozygous mutation was not present in the unaffected family members, and also 100 unrelated Chinese without cataracts serving as a control (Figure 3).

### Linkage and haplotype analysis

Genescan and linkage analysis were carried out by using 10 microsatellite markers flanking the *CRYBA3/A1* gene, and positive results were obtained, including the maximum HLOD score 3.005 at D17S1800 (α=1.000; Table 3). Haplotype analysis showed that the affected individuals in the family shared a common haplotype, with a region flanked by the markers D17S921 and D17S800 at 17p12–21.2 containing *CRYBA3/A1*.

**Table 3:** Multi-point linkage analysis between chromosome 17 DNA markers.

**Locus**	**Position (CM)**	**LOD**	**α**	**HLOD**
D17S799	31.960	1.485	1.000	1.485
D17S900/921	36.140	−6.484	0.000	0.000
D17S805	47.000	3.003	1.000	3.003
D17S1800	51.630	3.005	1.000	3.005
D17S1293	56.480	3.003	1.000	3.003
D17S1836	60.400	2.459	1.000	2.459
D17S800	62.010	−6.780	0.000	0.000
D17S1861	63.620	−6.603	0.000	0.000
D17S1817	103.530	−17.703	0.000	0.000

## Discussion

The genes which are reported to cause cataract-specific mutations include those of the crystallins, cytoskeletal proteins, membrane proteins, transcription factors, and chromatin modifying protein-4B [35]. Among the reported genes, the crystallins are of special interest because they encode the major proportion of water soluble structural proteins of the lens fiber cells. The human lens contains α-, β-, and γ-crystallins, of which the β-crystallins comprise the greatest part.

Thus far, three identified mutations in *CRYBA3/A1* gene have been associated with autosomal dominant cataract phenotypes of either a congenital or childhood nature. One is the IVS3+1 G>A mutation (position 474, GenBank M14303) which we reported [8,21,31], another is IVS3+1 G>C [30], and a third is a 3-bp deletion at positions 279–281 in exon 4 (279delGAG) [32,33]. Furthermore, the mutation IVS3+1 G>A has been found in 5 additional families. Kannabiran et al. [8] reported an Indian pedigree in which the phenotype was zonular cataracts with sutural opacities, Another two Indian pedigrees were reported by Devi et al. [31] with the phenotype of zonular lamellar opacities. An Australian family was reported by Burdon et al. [21] having the clinical features of Y-sutural opacities, mild opacification throughout the region of the fetal nucleus, and peripheral cortical dot opacities, and Gu et al. [34] identified a Chinese family with posterior polar cataracts, which was the first time this mutation was found in the Chinese population. Although the nuclear, sutural, and cortical cataracts were also found in the Australian family, the clinical features we observed were still distinctly different. First, in the family we studied, the nuclear cataracts in the affected individuals are progressive. Mild nuclear opacities with sutural opacities first appear at approximately 3 years of age, and progress thereafter. After 20 years of age, the nuclear opacities deteriorate so as to become lactescent, and thus become the main factor influencing the visual acuity. Second, the cortical cataracts in our case are also progressive. The fine, sand-like peripheral cortical opacities are observed just after 10 years of age, and progress with a trend of increasing and aggregating. After the age of 50 years they are almost curd-like. Fortunately, these cataracts in childhood are so mild that they exert little negative effect on visual development. After surgery, all of the patients achieved good visual acuity.

The related gene *CRYBA3/A1* is located in 17q11.2 (GeneID: 1411), which is a member of crystallin family, encoding two proteins (βA3-crystallin and βA1-crystallin) from a single mRNA. The latter protein is 17 amino acids shorter than βA3-crystallin and is generated by use of an alternate translation initiation site. Seven protein regions exist in βA1/A3-crystallin: four homologous motifs, a connecting peptide, and NH_2_- and COOH-terminal extensions. Each motif is a Greek key of four β-strands and consists of approximately 40 amino acid residues. The *CRYBA3/A1* gene consists of six exons [36]. In the *CRYBA3/A1* gene, the four Greek key motifs are approximately encoded by exons 3 to 6 [37]. Splice-site mutations were reported to result in exon skipping, activation of cryptic splice sites, creation of a pseudo-exon within an intron, or intron retention, among which exon skipping is the most frequent outcome [38]. The donor splice site of intron 3 of *CRYBA3/A1* (position 474) is the first nucleotide in the invariant GT dinucleotide of the 59 splice junction consensus sequence [30]. Due to the mutation IVS3+1 G>A, as we previously reported, the very next codon within the retained intron 3 would be a UGA stop site, which mimics a missense mutation at the protein level. Although the surveillance of mRNAs for transcripts that cannot be completely translated leads to their rapid degradation by nonsense-mediated decay (NMD) [39], transcripts of the IVS3+1 G>A of *CRYBA3/A1* probably escape the NMD pathway, and the premature termination codon (PTC) would cause truncation of the βA1/A3-crystallin immediately after the first motif. Since the Greek key motifs are formed with the fourth strand of the first motif being provided by the second motif and vice versa, without the second motif encoded by exon 4, it would not be possible to form even a single Greek key structure [40]. Due to the loss of exons 3 and 4 in *CRYBA3/A1* mRNA, a βA1/A3-crystallin species is suggested which contains only the COOH-terminal globular domain [8]. Gupta et al. [41] constructed eight deletion mutants of βA1/A3-crystallin and found that the deletion of exon 3 and exon 4 caused major structural instability, leading to the insolubilization of βA1/A3-crystallin. However there is as yet insufficient research in this field, and the nature of the complex mechanism remains to be determined.

In conclusion, we have identified a progressive form of congenital cataracts associated with the IVS3+1 G>A mutation of *CRYBA3/A1* in a Chinese family. This is the first report to relate this mutation to progressive cataracts. This study highlights the physiologic importance of βA1/A3-crystallin and supports the role of *CRYBA3/A1* in human cataract formation.
